# The hydrocortisone-responsive urinary metabolome of premature infants

**DOI:** 10.1038/s41390-023-02610-5

**Published:** 2023-05-03

**Authors:** Dara Torgerson, Miguel Guardado, Martina Steurer, Cheryl Chapin, Ryan D. Hernandez, Philip L. Ballard

**Affiliations:** 1 Epidemiology and Biostatistics, University of California San Francisco, San Francisco, CA USA; 2 Pediatrics, University of California San Francisco, San Francisco, CA USA; 3 Bioengineering and Therapeutic Sciences, University of California San Francisco, San Francisco, CA USA

## Abstract

**Background:**

Extremely premature infants are at risk for circulatory collapse or respiratory failure that are often treated with hydrocortisone (HC); however, there is no information on the metabolic consequences of this therapy.

**Methods:**

Longitudinal urine samples from infants <28 weeks gestation in the Trial of Late Surfactant were analyzed by untargeted UHPLC:MS/MS. Fourteen infants who received a tapering course of HC beginning at 3 mg/kg/day for ≥9 days were compared to 14 matched control infants. A secondary cross-sectional analysis by logistic regression used urines from 314 infants.

**Results:**

Of 1145 urinary metabolites detected, abundance of 219, representing all the major biochemical pathways, changed at *p* < 0.05 in the HC-treated group with 90% decreasing; 3 cortisol derivatives increased ~2-fold with HC therapy. Only 11% of regulated metabolites remained responsive at the lowest HC dose. Regulated metabolites included two steroids and thiamin that are associated with lung inflammation in infants. HC responsiveness was confirmed in 57% of metabolites by cross-sectional analysis.

**Conclusions:**

HC treatment of premature infants influenced in a dose-dependent manner abundance of 19% of identified urinary metabolites of diverse biochemical systems, primarily reducing concentrations. These findings indicate that exposure to HC reversibly impacts the nutritional status of premature infants.

**Impact:**

- Hydrocortisone treatment of premature infants with respiratory failure or circulatory collapse alters levels of a subset of urinary metabolites representing all major biochemical pathways.
- This is the first description of the scope, magnitude, timing and reversibility of metabolomic changes in infants in response to hydrocortisone, and it confirms corticosteroid regulation of three biochemicals that are associated with lung inflammatory status.
- The findings indicate a dose-dependency of hydrocortisone for metabolomic and anti-inflammatory effects, that prolonged therapy may lower the supply of many nutrients, and that monitoring concentrations of cortisol and inflammation markers may be a useful clinical approach during corticosteroid therapy.

## Introduction

Extremely low gestational age newborns are developmentally immature and at high risk for various disorders that include circulatory collapse, as manifest by hypotension and oliguria, and respiratory failure that can lead to bronchopulmonary dysplasia (BPD). The pathogenesis of respiratory failure is multifactorial, involving inflammation and oxidative stress imposed upon structurally and functionally immature lungs.^1–3^ Both circulatory collapse and respiratory failure in premature infants are related in part to developmental immaturity of the hypothalamic–pituitary–adrenal axis.^4^

Glucocorticoids are known to enhance vascular tone, stimulate fetal lung structural and functional development, and have strong anti-inflammatory effects in the developing lung,^5–7^ providing the rationale for cortisol (hydrocortisone (HC)) treatment of selected infants in Neonatal Intensive Care Units. HC is often used for hypotension that is refractory to volume expansion and ionotropic/vasopressor support, and therapy is effective within a few hours.^3^ Dexamethasone is one of the few therapies shown to improve respiratory status and outcome in premature infants with respiratory failure; however, adverse outcomes such as intestinal perforation, growth impairment and neurological sequelae are increased depending on dose, duration and timing of treatment.^8,9^ Low-dose HC therapy (1–2 mg/kg/day) is viewed as a safer approach and a meta-analysis indicated a significant reduction in BPD without adverse effects other than increased intestinal perforation in combination with indomethacin.^10^ By contrast, later (initiated at 7–28 days), higher-dose HC treatment (4–5 mg/kg/day) of infants receiving mechanical ventilation resulted in earlier extubation but did not impact survival without moderate or severe BPD.^11,12^ Because glucocorticoids have multiple differentiation, metabolic and anti-inflammatory effects, there is a need to better describe biochemical responses in infants to optimize the benefit:risk ratio.

Untargeted metabolomic analysis involves separation and identification of low molecular weight chemicals by ultrahigh-performance liquid chromatography and mass spectrometry (UHPLC:MS/MS) to provide quantitative information on hundreds of metabolites in a biofluid, including infant tracheal aspirate.^13–15^ Using this approach, metabolic effects of glucocorticoids have been described in adults and in 3 studies with infants receiving potent synthetic corticosteroids; however, there is no information available on metabolic perturbations in infants receiving relatively low-dose HC.^16–19^

We recently reported metabolomic data from a dose escalation study (Steroids and Surfactant in ELGANs (SASSIE)) to determine the optimal dose (and frequency) of instilled budesonide, a synthetic corticosteroid, suspended in surfactant to treat intubated infants at high risk for BPD.^20^ In this time course analysis, there was a dose-response relationship for altered levels of budesonide-regulated blood metabolites, and selected regulated metabolites were associated with the level of lung inflammation.

In the current study, we used untargeted metabolomic analysis of urine to investigate time-dependent responses to low-dose HC treatment in premature infants of the Trial of Late Surfactant (TOLSURF).^21^ We also expanded our comparisons to a larger number of infants using a nested, cross-sectional design. Our findings provide new information on specific biochemicals and pathways affected by HC and generate insights into the clinical use of HC in ill premature infants.

## Methods

### Clinical studies

TOLSURF (ClinicalTrials.gov, NCT01022580) was a blinded, randomized, sham-controlled trial performed between 2010 and 2013 at 25 US centers and designed to assess the effects of late surfactant treatments on respiratory outcome. The trial design, infant characteristics and effects of late surfactant treatment have been reported.^21^ A total of 511 extremely low gestational age (<28 weeks) infants receiving mechanical ventilation at 7–14 days were enrolled and received either surfactant (calfactant/Infasurf) or sham installation every 1–3 days. Clinical information, including selected medications and respiratory parameters, was collected, and the respiratory severity score (RSS, FiO_2_ × mean airway pressure) was calculated. Survival without BPD did not differ between the treated and control groups at either 36 or 40 weeks postmenstrual age.

HC therapy was used in 180 of 512 TOLSURF infants (duration 20.1 ± 19.1 days, median 13.5, range 1–101 days with 246 courses of treatment) for the clinical indications of hypotension refractory to dopamine and dobutamine (25.6% of courses), suspected adrenal insufficiency (27.4%), to prevent BPD (32.9%), to facilitate extubation (9.8%) or other reason (4.5%). The recommended dosing regimen was 3 mg/kg/day in two divided doses for 3 days, 1.5 mg/kg/day for 3 days, 0.5 mg/kg/day for 3 days and then off HC if tolerated or continued at an effective dose.

The Prematurity and Respiratory Outcomes Program (PROP, NCT01435187) was an observational study performed between 2010 and 2013 at 8 US centers and designed to collect clinical data and biospecimens for analyses of factors related to respiratory outcomes. A total of 835 infants ≤28 weeks were enrolled at 1–7 days with characteristics as previously described.^22^ PROP infants with metabolomic data received HC in divided doses at a starting dose of 5.2 ± 0.2 (mean/SD) mg/kg/day tapering to 0.5 ± 0.2 mg/kg/day, with a dose of 2.4 ± 1.8 mg/kg/day at urine collection on day 28.

The research protocols for both TOLSURF and PROP were approved by the Institutional Review Boards of the participating institutions, and a parent of each infant provided written informed consent.

Urine samples were collected from most infants of both TOLSURF (≤4/week for up to 8 weeks) and PROP (days 3, 7, 14 and 28) using cotton balls in the diaper for 4–8 h and were stored at –70 °C. The primary longitudinal metabolomic analysis was performed on 302 available urine samples from 28 TOLSURF infants at 15 different institutions—14 HC-treated and 14 matched (by birthweight, sex, race and RSS) controls with no corticosteroid exposure. The secondary cross-sectional analysis used urines collected at 23–32 (27.6 ± 2.2) days from 171 TOLSURF infants (23 receiving HC treatment) and at 26–30 (28.1 ± 0.9) days from 143 PROP infants (41 treated with HC).

### Metabolomic analysis

Untargeted metabolomic analysis of urines used UHPLC:MS/MS performed by Metabolon Inc (Morrisville, NC) as described.^17^ The dataset for the longitudinal study comprised a total of 1145 compounds of both known identity (named) and unnamed (24.8%) chemicals. Raw area counts for each biochemical were adjusted for any inter-assay (batch) variability and then were rescaled setting the median value for each chemical to equal 1 and further normalized to urine osmolality. Values below the limit of detection, if any, were imputed using the minimum detectable value for each chemical.

To determine the fold-change in metabolite level after starting HC treatment, we used normalized data and determined the ratio of concentration in the first sample collected at 2–10 days after starting HC (post) compared to a sample at 0–2 days before starting treatment (pre). The same approach was used to determine the change in metabolite level with stopping HC (off/on analysis). Comparable time course samples were selected for control infants. To determine the relative abundance of urinary cortisol derivatives, we used data for area under the peak and normalized values to cortisone 21-sulfate (the most abundant metabolite) for each sample.

### Statistical analysis

Analysis of data in the time course study used Student’s paired *t*-test comparing normalized concentrations at the two time points for each infant and ANOVA with Tukey’s HSD test for comparing HC responses by biochemical pathway. Data are presented as mean ± SD or as median and interquartile range. Clinical characteristics of HC-treated vs control infants used Student’s unpaired *t*-test and Fisher’s exact test. Principal component analysis (PCA) was performed in R after removing metabolites with >30% missing/imputed data in the combined dataset. Cross-sectional analysis of HC treatment and metabolite abundance at day 28 were compared using logistic regression for infants on vs. off HC, adjusting for birthweight, sex, multiple births, and maternal race/ethnicity. Comparisons were performed within each study and then combined across TOLSURF and PROP using a weighted meta-analysis to generate an odds ratio value.

## Results

### Study group

This report focuses on the longitudinal analysis of 151 urine samples from 14 HC-treated infants over a mean interval of 36 ± 11 days (range 19–54) and 151 urine samples for matched controls over 33 ± 6 days (range 21–44). Demographics and clinical parameters were similar between groups except for a later age at the transition to oral feeds in HC-treated infants (Table 1). All infants except three were receiving total parenteral nutrition (TPN) during the time period of urine collections for analysis of metabolite abundance; later transition to full oral feeds occurred over about 1 week.

**Table 1:** Characteristics of HC-treated and matched control infants.

	HC-treated (*n* = 14)	Control (*n* = 14)	*p*
Gestational age (weeks)	24.8 ± 0.9	25.2 ± 0.9	NS
Birthweight (g)	706 ± 158	716 ± 156	NS
Sex (male/female)	9/5	9/5	NS
Race/ethnicity (W/AA/His)	6/4/4	7/5/1	NS
Antenatal corticosteroid (no/yes)	0/14	2/12	NS
Average RSS (days 7–14)	4.1 ± 1.3	3.8 ± 1.3	NS
BPD36 weeks (no/yes)	6/8	5/9	NS
BPD40 weeks (no/yes)	8/6	8/6	NS
Late surfactant (no/yes)	9/5	8/6	NS
Age start HC (days)	16.0 ± 6.6	–	
Days with urine samples	36 ± 11	34 ± 7	NS
Age at full oral feeds (days)	35.1 ± 6.1	23.3 ± 4.9	0.0001

Data are mean ± SD with *p* value by unpaired *t*-test or Fisher’s exact test. There are no differences in demographics or clinical parameters between groups except for later age at full oral feeds for HC-treated infants.

*W* white, *AA* African-American, *His* Hispanic.

### Excreted cortisol derivatives

**Fig. 1:**
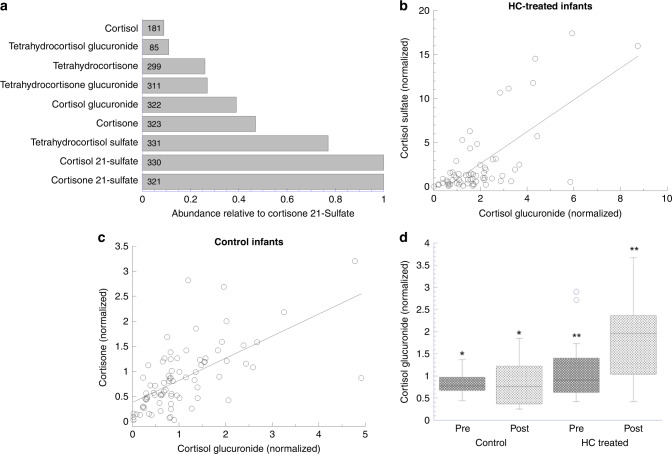
Excreted cortisol and cortisol derivatives and effect of HC treatment on cortisol glucuronide. **a** Relative abundance of urinary cortisol and derivatives normalized to cortisone 21-sulfate. Total urine samples = 332; number of urines with steroids detected are shown in each bar. **b** Cortisol glucuronide vs cortisone sulfate in HC-treated infants. *n* = 64 for 14 infants while on HC (range 15–63 days); *r* = 0.70, *p* = 8.7e–11 by linear regression. **c** Cortisol glucuronide vs cortisone urinary levels in control infants. *n* = 80 for 14 infants during a comparable time period to HC-treated infants (range 10–45 days); *r* = 0.69, *p* = 4.0e–10. **d** Comparison of cortisol glucuronide abundance in HC-treated and control infants. Data are for urine samples collected over 10 days before starting HC (Pre) and 10 days during HC therapy (post). Levels in HC-treated infants increased 1.9- (mean)/2.6-fold (median) and were 2.0-fold higher vs control infants. *p = 0.31; **p = 0.006; *n* = 14/14 infants and 44/53 (post/pre) samples in control infants and 52/38 in HC-treated infants.

We found detectable levels of nine corticosteroids related to cortisol and cortisone in most urine samples. The sulfated derivatives of cortisol and cortisone were most abundant with relatively lower levels of glucuronide derivatives; unmodified cortisol was detected in only 55% of the samples, whereas cortisone was present in 97% of samples (Fig. 1a). On examining relative levels of the different steroids, the highest correlations were observed between cortisol glucuronide and cortisone sulfate (*r* = 0.70) in HC-treated infants (Fig. 1b) and between cortisol glucuronide and cortisone (*r* = 0.69) in control infants (Fig. 1c). To examine the response of different cortisol derivatives to HC, we compared the mean concentration over 10 days on HC vs a comparable interval for control infants; significant differences (*p* < 0.02) occurred for cortisol glucuronide (HC vs control 1.9-fold, *p* = 0.006 Fig. 1d), cortisone (1.9-fold), and tetrahydrocortisol sulfate (2.0-fold). Based on its high detection rate in urines and highly significant differences for both before/after HC treatment and abundance in HC-treated vs control infants, we selected cortisol glucuronide as the best indicator of the concentrations of circulating cortisol.

### HC-responsive metabolites by post/pre analysis

Untargeted metabolomic analysis of 302 urine samples detected 1145 different metabolites with 76% chemically identified (named). We identified by post/pre analysis a subset of urinary metabolites that changed in concentration with the onset of HC treatment. This individual-infant approach utilizing longitudinal data minimizes the impact of differences between infants in clinical status, medications and respiratory support that impact metabolic status. By paired *t*-test, there were 219 metabolites (named and unnamed, with 5 detected in <70% of samples) with *p* < 0.05 in the HC-treated group and 14 of these metabolites with *p* < 0.05 in controls. Thus, at a significance of *p* < 0.05, commencement of HC treatment affected up to 19% of reliably detected urinary metabolites.

Table 2 lists 44 named biochemicals with *p* < 0.01 for response to HC; the mean post/pre values ranged from 0.48 to 3.26 with the concentration of 34 metabolites decreased and 10 increased. The list includes 17 amino acids/peptides, 15 lipids with 9 representing steroids or steroid precursors, 4 carbohydrates, 4 cofactors/vitamins (2 nicotinamide-related) and 2 xenobiotics. In control infants, 5 of these biochemicals had a post/pre value <1 with a *p* value <0.05 (none at *p* < 0.01); all 5 changed in the same direction as with HC treatment, suggesting a developmental influence for levels of these metabolites. A complete list of post/pre values is given in Supplementary Table S1.

**Table 2:** Post/pre change in 44 named metabolites at *p* < 0.01 with HC treatment.

	Post/pre HC-treated			Post/pre control		
Chemical name	Mean	SD	*p*	Mean	SD	*p*
17α-Hydroxypregnanolone glucuronide	0.48	0.25	0.000003	0.79	0.55	0.218
16α-Hydroxy DHEA 3-sulfate	0.63	0.87	0.00047	0.93	0.69	0.253
3-Hydroxy-3-methylglutarate	0.77	0.30	0.00052	1.16	0.65	0.701
N-acetyl-3-methylhistidine	0.64	0.32	0.00082	0.80	0.32	0.044*
Trigonelline (N’-methylnicotinate)	0.68	0.26	0.00086	1.05	0.49	0.701
6-Sialyl-N-acetyllactosamine	0.57	0.30	0.00092	1.04	0.70	0.475
11-Ketoetiocholanolone sulfate	0.69	1.28	0.00100	0.78	0.31	0.045*
Cortisol glucuronide	1.99	1.06	0.00169	1.04	0.70	0.873
Cyclo(pro-hydroxypro)	0.54	0.30	0.00189	1.01	0.53	0.740
Androsteroid monosulfate	0.68	0.68	0.00205	0.89	0.45	0.341
Myo-inositol	0.70	0.68	0.00226	1.36	1.21	0.460
Tigloylglycine	2.80	1.49	0.00231	1.24	0.76	0.975
Phenylacetyltaurine	0.55	0.60	0.00248	0.87	0.84	0.051
Mevalonolactone	0.76	0.29	0.00271	0.95	0.61	0.733
2-Pentenoylglycine	3.26	2.58	0.00296	1.06	0.64	0.722
11β-Hydroxyandrosterone glucuronide	0.85	1.82	0.00305	1.15	1.23	0.630
Androstenediol (3a, 17a) monosulfate	0.59	0.55	0.00310	1.74	2.30	0.400
Lyxonate	0.67	0.35	0.00323	1.05	0.71	0.495
2-Pyrrolidinone	0.68	0.31	0.00332	0.97	0.47	0.418
Furaneol sulfate	0.71	0.37	0.00365	0.96	0.55	0.818
5-(Galactosylhydroxy)-L-lysine	0.55	0.31	0.00420	1.30	0.97	0.630
Isobutyrylglycine	3.05	2.33	0.00422	0.99	0.35	0.694
Phenylacetylthreonine	0.71	0.92	0.00445	0.75	0.54	0.045*
Pregnenetriol sulfate	0.86	0.82	0.00481	1.23	0.58	0.269
Diacetylspermidine	0.74	0.32	0.00516	0.95	0.46	0.629
Delta-CEHC sulfate	0.76	0.38	0.00562	0.73	0.30	0.016*
4-Acetamidobutanoate	0.70	0.32	0.00567	0.98	0.49	0.481
1-Methylnicotinamide	0.75	0.26	0.00631	1.00	0.38	0.414
2-Methylbutyrylglycine	2.99	2.56	0.00633	1.62	1.08	0.092
o-Cresol sulfate	0.76	0.80	0.00688	0.74	0.38	0.014*
Symmetric dimethylarginine	0.68	0.32	0.00690	0.98	0.73	0.602
Isovalerylglycine	2.95	2.68	0.00698	1.13	0.51	0.763
Erythronate	0.75	0.22	0.00708	0.98	0.37	0.552
Indoleacetylglutamine	3.04	2.17	0.00709	1.45	0.93	0.265
3’-Sialyllactose	0.61	0.31	0.00741	1.17	0.88	0.502
Glucuronide of C_8_H_14_O_2_	0.72	0.39	0.00747	2.33	3.97	0.413
3-Hydroxyadipate	0.61	0.40	0.00750	1.00	0.58	0.251
3-Methylcrotonylglycine	4.10	4.71	0.00774	1.51	1.37	0.750
Tetrahydrocortisone	3.17	1.71	0.00786	1.38	1.17	0.500
3-Methoxy-4-hydroxyphenylglycol	0.89	0.87	0.00809	1.00	0.64	0.691
Isovalerylglutamine	4.52	5.95	0.00814	1.29	1.21	0.824
Nicotinamide riboside	0.83	0.53	0.00880	1.08	0.36	0.755
Glycerophosphoserine	0.68	0.28	0.00887	1.28	1.51	0.298
Orotidine	0.67	0.31	0.00972	0.90	0.48	0.142

Results are for 44 named (identified) biochemicals with *p* < 0.01 and detectable levels in >70% of all samples. Data for an additional 16 unnamed metabolites and 3 metabolites (glycerolphosphoinositol, furosemide, vanillate glucuronide) with <70% detection rate in the post/pre samples are not shown. Pre value for HC-treated infants is abundance of metabolite in a urine sample obtained 0–2 days before starting HC and post value is abundance in the first urine sample available at least 2 days after starting HC. The same approach was used for selecting samples at comparable ages in control infants. *p* value determined by paired *t*-test comparing normalized metabolite abundance in the post and pre samples for each infant.

*Five biochemicals with post/pre *p* value <0.05 in control infants.

**Fig. 2:**
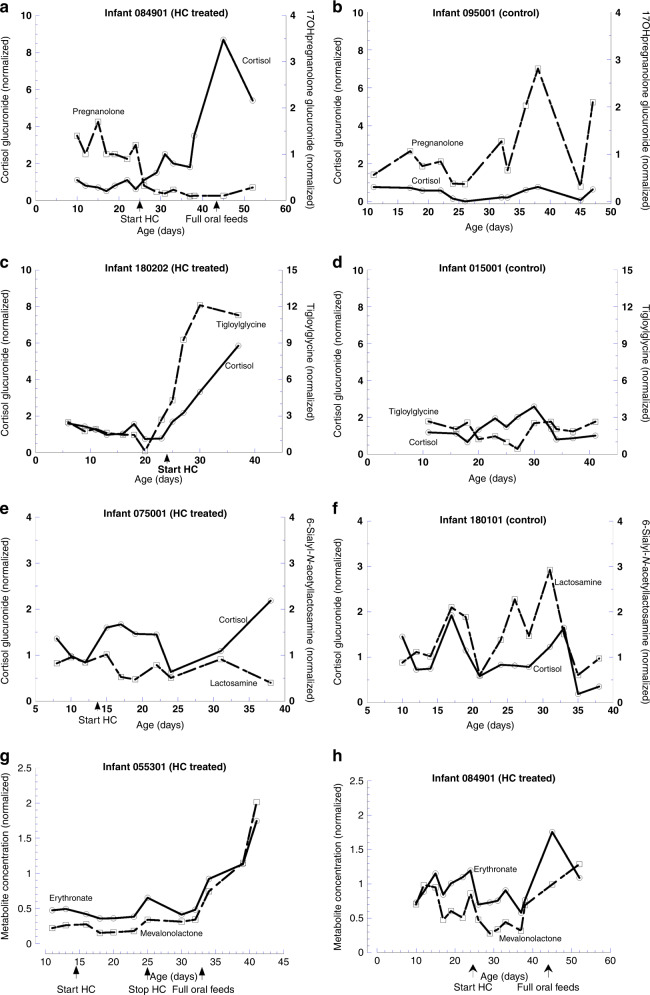
Examples of urinary metabolite abundance over time in HC-treated and control infants. **a** 17hydroxypregnanolone glucuronide in an infant treated with HC days 25–81 and receiving full oral feeds at day 44; **b** 17hydroxypregnanolone glucuronide in a control infant; **c** tigloylglycine, a metabolite of isoleucine, in a HC-treated infant days 24–52; **d** tigloylglycine in a control infant; **e** 6-sialyl-N-acetyllactosamine, an oligosaccharide, in a HC-treated infant days 14–48; **f** 6-sialyl-N-acetyllactosamine in a control infant. **g** Concentrations of erythronate and mevalonolactone in an infant receiving a 10-day course of HC; levels decrease on starting HC and increase 1.6- and 1.9-fold by off/on analysis on stopping HC. **h** Concentrations of erythronate and mevalonolactone remain lower until transitioning to oral feeds in an infant on HC days 25–81.

Examples of metabolite levels in individual infants illustrate temporal changes and increased cortisol glucuronide with HC treatment (Fig. 2). The concentration of 17hydroxypregnanolone glucuronide decreased by two-thirds to a new plateau level one day after starting HC (Fig. 2a), whereas an increase over time occurred in a control infant (Fig. 2b). Tigloylglycine abundance increased after HC, reaching a 6-fold higher plateau level after 5 days (Fig. 2c) with no consistent change in control (Fig. 2d). 6-sialyl-N-acetyllactosamine decreased by ~40% 3 days after starting HC with ~2-fold increase in cortisol glucuronide (Fig. 2e); in the control infant (Fig. 2f), concentrations of both metabolites were variable over time. These results indicate altered metabolite levels ≤2 days after starting HC.

We used an off/on paired sample approach to determine changes in metabolite abundance with cessation of HC therapy, expecting a reciprocal change in concentration to that occurring on starting HC. This correlation would occur only for metabolites responsive to the lowest dose (0.5 mg/kg/day) of HC in the tapering treatment regimen used in TOLSURF (Methods). Urine samples were available after stopping HC for 7 of the 14 treated infants, and we used samples from the other 7 infants who remained on HC as controls for continuing HC exposure. Of the 219 metabolites with post/pre values of *p* < 0.05 (Supplementary Table S1), 23 metabolites (10.5%) had an off/on change (mean 3.2 ± 1.5-fold) in concentration at *p* < 0.05 that was in the opposite direction to the change on starting HC (Table 3). Two examples are erythronate and mevalonolactone that illustrate a reversal of suppression on stopping HC after 10 days and continued suppression in an infant receiving a long course of HC (Fig. 2g, h, respectively). The 23 metabolites represented 7 different Major Pathways. All data for the off/on analyses are given in Supplementary Table S2. These results indicate that only a subset of HC-responsive metabolites has a sustained response at the lowest levels of HC used in this study.

**Table 3:** Metabolites responsive to both initiation and cessation of tapering dose HC treatment.

		Off/on HC treatment analysis						Post/pre HC		
Chemical name	Super pathway	HC stopped (*n* = 7)			HC continued (*n* = 7)			(*n* = 14)		
		Mean	SD	*p*	Mean	SD	*p*	Mean	SD	*p*
Leucylglycine	Peptide	4.83	4.80	0.007	1.62	1.31	0.7454	0.65	0.79	0.031
Isoleucine	Amino acid	2.28	1.60	0.017	1.23	1.02	0.8410	0.87	0.40	0.034
2-Hydroxyhippurate	Xenobiotics	4.24	4.03	0.020	1.20	0.55	0.7557	0.63	0.69	0.022
Isocitrate	Energy	6.12	3.89	0.024	4.27	5.28	0.2658	0.65	0.60	0.035
Isovalerylglutamine	Amino acid	0.51	0.33	0.024	1.67	3.26	0.0481	4.52	5.95	0.008
3-Methylcrotonylglycine	Amino acid	0.62	0.37	0.024	1.03	0.21	0.7320	4.10	4.71	0.008
X-24332		4.72	4.28	0.028	3.82	7.02	0.4830	0.68	0.47	0.013
X-12101		2.95	1.48	0.029	1.30	0.98	0.4555	0.75	0.37	0.008
Phenylalanylglycine	Peptide	2.12	1.00	0.033	1.50	0.86	0.2826	0.75	0.51	0.033
X-10458		2.59	1.47	0.033	1.17	0.84	0.9101	0.73	0.30	0.004
X-18887		1.89	0.86	0.034	1.35	0.77	0.4101	0.69	0.46	0.013
X-24341		4.03	2.37	0.034	3.21	5.76	0.5111	0.68	0.67	0.011
Pro-hydroxy-pro	Amino acid	1.98	0.95	0.035	1.65	1.01	0.1605	0.75	0.44	0.031
Leucylhydroxyproline	Peptide	3.60	2.10	0.036	2.40	1.98	0.2508	0.73	0.53	0.036
X-17842		2.21	1.39	0.041	1.08	0.72	0.8538	0.75	0.34	0.011
3-Methylcytidine	Nucleotide	1.42	0.46	0.041	1.35	0.64	0.2253	0.81	0.24	0.018
Homocitrate	Energy	2.74	1.73	0.041	1.66	1.28	0.2430	0.79	0.40	0.012
X-23665		4.56	5.17	0.044	1.29	0.96	0.6991	0.79	0.86	0.028
Mevalonolactone	Lipid	2.88	1.91	0.045	1.70	1.57	0.4946	0.76	0.29	0.003
Glycerate	Carbohydrate	2.84	2.46	0.045	1.96	2.15	0.3226	0.86	0.24	0.043
Erythronate	Carbohydrate	1.53	0.54	0.047	1.22	0.45	0.2425	0.75	0.22	0.007
N-methylproline	Amino acid	6.74	6.71	0.048	5.78	12.17	0.4349	0.71	0.46	0.012
X-23678		3.41	2.18	0.048	2.74	3.54	0.3114	0.76	0.54	0.006

Data are for 23 chemicals with *p* < 0.05 in both off/on analysis after stopping HC and in post/pre on starting HC. Five chemicals with a change in the same direction for off/on (stop HC) and post/pre (start HC) are not shown. *p* values by paired *t*-test of normalized chemical abundance. Only 10.5% of metabolites regulated at *p* < 0.05 on starting HC remain responsive at the lowest dose of HC before stopping treatment.

**Fig. 3:**
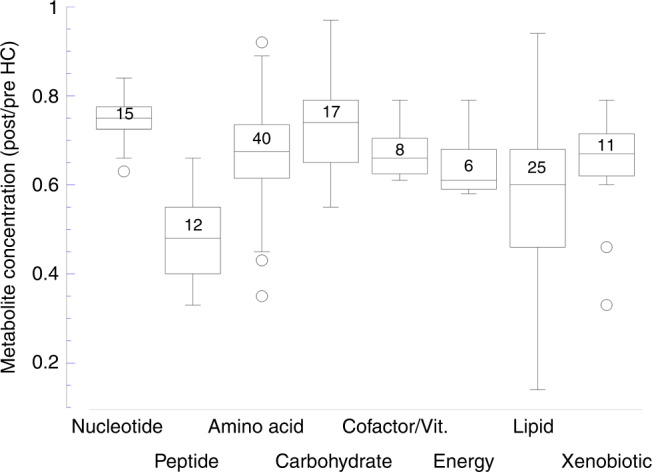
HC suppression in post/pre analysis of named metabolites by Super Pathway assignment. Box plots with number of suppressed metabolites in each pathway. Significance between pathways by ANOVA with Tukey’s HSD: nucleotide vs lipid *p* = 0.0008, peptide vs amino acid *p* = 0.0003, peptide vs cofactor/vitamin *p* = 0.022, carbohydrate vs lipid *p* = 0.003. HC suppresses metabolites in each of 8 Super Pathways with significant differences in magnitude.

Overall, concentrations decreased for 90.4% of HC-responsive (*p* < 0.05) metabolites. Post/pre values increased for 12 named endogenous biochemicals (mean 2.02 ± 0.31 fold-change, range 1.68–2.72), and these included cystine, 2 cortisol derivatives, and 9 acyl glycine/glutamine biochemicals. By Super Pathway assignment, the mean degree for suppressed metabolites ranged from 0.48 (peptide) to 0.75 (nucleotide) with statistically significant differences between some pathways (Fig. 3). Full results by Super Pathway are given in Supplementary Table S3.

In an enrichment analysis, regulated metabolites represented all 8 Super Pathways as classified by Metabolon (amino acid, carbohydrate, cofactor/vitamin, energy, lipid, nucleotide, peptide, xenobiotic) and 50 of a total of 100 Sub Pathways. Of 17 Sub Pathways containing at least 4 biochemicals, the percentage of metabolites responsive to HC was enriched for 2 categories at *p* < 0.05 compared to all detected metabolites: androgenic steroids (fold enrichment 3.3, *p* = 0.005) and aminosugar metabolism (3.1-fold, *p* = 0.037). This generalized response to HC across the metabolome is consistent with the known diverse metabolic effects of corticosteroids.

Concentrations of seven biochemicals related to vitamins, which are of clinical and therapeutic interest, were affected by HC at *p* < 0.05 (Table 2 and Supplementary Table S1): retinol (vitamin A, post/pre 2.49, *p* = 0.029), thiamin (vitamin B1, post/pre 0.86, *p* = 0.015), 4 nicotinamide-related biochemicals (precursors, metabolites or versions of niacin, vitamin B3—post/pre 0.68-0.83, *p* < 0.01), and delta-CEHC sulfate (oxidized tocopherol, vitamin E—post/pre 0.76, *p* = 0.006). Vitamins A, B1, B3 and E are components of parenteral nutrition, which all infants received; four infants also received 16–25 days of intramuscular vitamin A treatment but there was no impact on the HC response (data not shown).

### HC-responsive metabolites by cross-sectional comparison

To further assess which metabolites are influenced by HC and to capture population-level variation in the metabolic response, we analyzed separate metabolomic data for single urine samples that were collected at day 23–30 from infants of both TOLSURF (*n* = 23 HC-treated, 135 no treatment) and PROP (*n* = 41 HC-treated, 102 no treatment) cohorts. HC-treated PROP infants in the cross-sectional comparison had lower gestational age and birthweight and remained longer on TPN compared to untreated infants, and HC-treated TOLSURF infants had higher RSS (Supplementary Table S4).

**Fig. 4:**
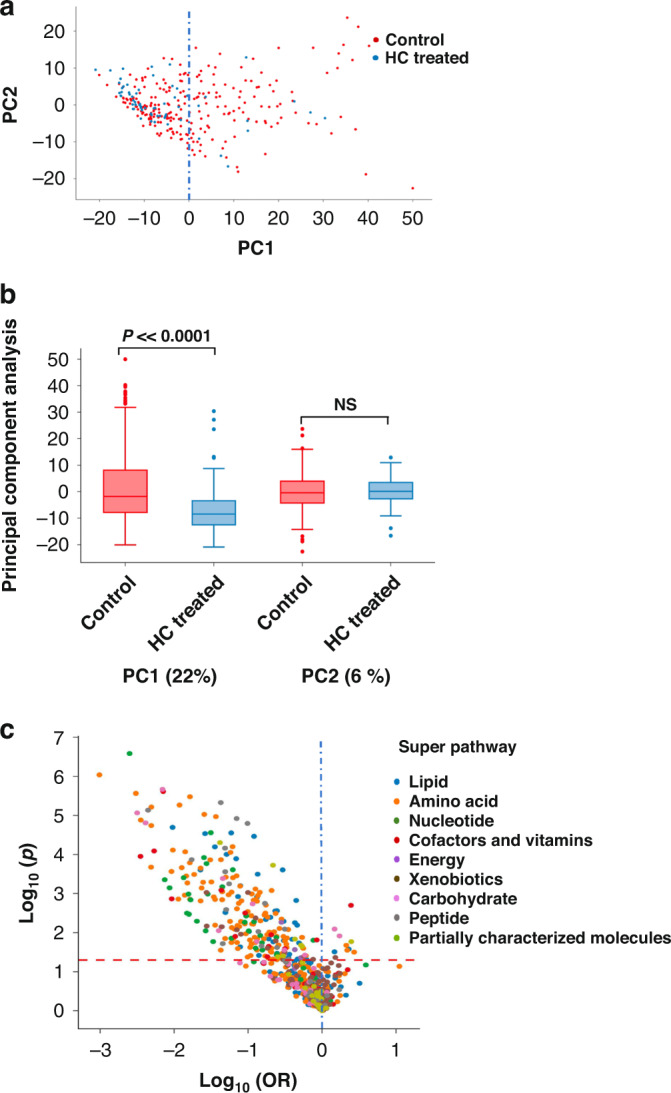
Effect of HC on the urinary metabolome by cross-sectional analysis. **a** PCA of 1010 metabolites with <30% imputed values. PC1 accounts for 22% of the variance in metabolite abundance between HC-treated and untreated infants with the majority reduced by HC. **b** Box plot. Significance of PC1 (*p* = 8.3e–7) and not PC2 (*p* = 0.65) in PCA analysis. **c** Volcano plot. Significant differences (above dashed red line) with HC are primarily negative (left of 0) and include metabolites of all super pathways.

Exploratory approaches to data analysis are shown in Fig. 4. Using PCA, global metabolomic status (principal component 1, PC1) varies significantly by HC treatment (22%, Fig. 4a, b), whereas PC2 explained 6% (NS). The Volcano plot of Fig. 4c shows individual metabolites influenced by HC: most of the responses were negative (lower metabolite level) and involved metabolites of all major pathways, as also found in the post/pre study.

We used logistic regression for each cohort of infants, adjusting for birthweight, sex, maternal race/ethnicity and feeding status (TPN vs oral feeds), followed by a meta-analysis. Of 1424 metabolites, 227 had a *p* < 0.01 for HC-treated vs no treatment (Supplementary Table S5). Comparing to the 53 metabolites with an HC post/pre response at *p* < 0.01, the meta-analysis p value was <0.01 for 20 (38%), and <0.05 for 30 (57%) with the direction of change with HC the same for each metabolite. Thus, a response to HC in the time course study was confirmed by cross-sectional analysis for many but not all responsive metabolites.

## Discussion

In this study, we examined changes in the urinary metabolome in response to HC treatment in premature infants with respiratory failure and/or hypotension. Using a longitudinal analysis, we found that urinary cortisol derivatives increased and levels of ~19% of detected metabolites, representing all major metabolic pathways, changed concurrently with most decreasing in concentration. These results represent the first information to our knowledge on global metabolic consequences of HC therapy of the ill premature infant and demonstrate that even a relatively low dose of HC has a variety of metabolic consequences that could impact infant nutrition, growth and medication requirements in addition to desired anti-inflammatory and circulatory stability responses.

As noted by Heckmann et al.,^23^ collection of infant urine from diapers (and nappies) provides a non-invasive approach to assessing and monitoring excreted metabolites. They found that excretion of total corticosteroids (*n* = 13) decreased ~2-fold over the first 4 weeks postnatally with considerable variability as expected for ill premature infants. In control infants of our study, there was no consistent pattern over time except for an increase in four cortisol and cortisone derivatives on transition from TPN to oral feeds. We found only low or undetectable concentrations of free cortisol, whereas more soluble, biologically inactive steroid derivatives (sulfate and glucuronide) were abundant. Cortisone was relatively abundant, which contrasts with results for adult urines,^24^ likely reflecting higher levels of 11-beta hydroxysteroid dehydrogenase (Type 2) and circulating cortisone in infants. In addition, sulfated derivatives of cortisol and cortisone were abundant in infant urines but not in the adult, consistent with major developmental changes in corticosteroid metabolism.^25^ Overall, our findings suggest that targeted measurements of selected urinary corticosteroids may be useful for monitoring adrenal status and response to HC therapy.

In TOLSURF, about a third of the HC-treated infants were treated for hypotension, which in many cases was refractory to vasopressor/ionotropic therapy. The blood pressure increase with HC in infants is often rapid (within hours), suggesting that the mechanism is likely non-genomic (i.e., direct glucocorticoid-protein interaction) in contrast to metabolic effects (delayed response secondary to altered gene expression), which predicts that different dose- and time-response properties may exist between vascular and metabolic responses.^26^ Because blood pressure measurements were not collected in TOLSURF, we could not compare metabolomic data and hemodynamic response; this clinical information is needed to determine optimal dosing and duration for the efficacy of HC therapy for circulatory collapse with minimal nutritional impact.

Another third of TOLSURF infants were treated with HC for the prevention of BPD. This indication for postnatal corticosteroids targets primarily anti-inflammatory effects, and benefit depends on the timing and dose of the steroid. Doses of HC in early initiation trials were 1–2 mg/kg/day and resulted in blood cortisol levels 66% higher than controls, which is similar to our observations for urine (~90% increase) after 3 mg/kg/day HC.^10^ The increased prevalence of adverse effects with dexamethasone vs HC most likely reflects the higher glucocorticoid exposure with dexamethasone, which has more pronounced and additional metabolic effects as observed with budesonide.^9,17^

There are limited published data for metabolomics responses to glucocorticoid exposure in infants. In two studies, Lewis et al. performed untargeted metabolomic analysis on urine samples that were collected before and 3–6 days after a tapering dose of systemic dexamethasone given to 10 premature infants in respiratory failure.^16,18^ Similar to our results with HC, they found that the predominant metabolite response to exogenous corticosteroid was decreased concentrations (13 of 18 biochemicals that changed at *p* < 0.055). Of the 18 metabolites, 13 were detected in our study with HC but only 3 were altered at *p* < 0.05. In part, this difference is likely due to higher corticosteroid exposure with dexamethasone compared to HC.

Previously, in the SASSIE study, we determined the time course of blood metabolic response in premature infants to three different doses of instilled budesonide, a potent glucocorticoid.^17^ Among 90 biochemicals with highly significant changes in concentration after exposure, 15 demonstrated a similar response at each of the budesonide doses (0.025, 0.05 and 0.1 mg/kg), indicating high sensitivity to glucocorticoid. These budesonide-regulated metabolites included four different derivatives of pregnanolone and nine other metabolites that were also detected in urine of HC-treated infants. The response to HC was in the same direction for six of the nine metabolites, and one of the three discrepancies was for cortisone, which increased in HC infants and decreased with budesonide as expected due to suppression of the HPA axis. The degree of suppression with HC was less than with low-dose budesonide (0.70 ± 0.11 vs 0.46 ± 0.17, *p* = 0.02). Thus, our current results are in generally good agreement with the previous data for exposure to lower-dose glucocorticoid but with smaller fold-changes. Based on the budesonide results, we would expect levels of additional metabolites to be altered by higher-dose HC regimens or treatment with dexamethasone.

Findings in this study and the previous SASSIE project provide new information on the properties and scope of glucocorticoid therapy in premature infants that are of interest for clinical management. First, both the number and magnitude of the biochemical responses vary with budesonide dose, and presumably as well with HC and dexamethasone, with some metabolites maximally altered at the lowest dose and others only changed at higher doses. Ideally, if an infant’s clinical condition allows, treatment dosage should be incrementally increased from a low dose and the desired clinical response (e.g., blood pressure) monitored, which would potentially allow lower doses to be used and avoid undesired metabolic responses (e.g., hyperlipidemia) that occur at higher doses.^17,27^ Second, most metabolic responses to corticosteroids have a lag time of 1–2 days. Accordingly, most physiological and clinical responses would not be expected until after that interval, and responses to a change in dose would similarly be delayed. Third, analysis after stopping HC indicated a response for about 10% of the HC-responsive biochemicals, suggesting that only a relatively small subset of metabolites remain responsive at the lowest HC dose (0.5 mg/kg). Thus, many metabolic effects and likely physiologic responses seen after starting HC at the dosing regimen used in TOLSURF are reversed before cessation of treatment, and conversely, the lowest therapeutic dose for most metabolic effects is >0.5 mg/kg in premature infants. Our results also suggest that infants receiving long-term administration of HC at doses >0.5 mg/kg may be at increased risk for adverse systemic effects. Fourth, most (90%) of the changes in metabolite levels with HC therapy were negative, indicating a reduced supply of specific nutrients and possible benefit from an increased dose of TPN for infants receiving a prolonged course of HC. Fifth, HC treatment was associated with changes in urinary levels of some vitamins and drugs. For example, abundance of four metabolites of niacin (vitamin B3) decreased by 17–32% with HC treatment, which may indicate a need to increase the dose of niacin supplementation with corticosteroid therapy.

Lung inflammation is considered a component of the pathogenesis of respiratory failure and BPD and is a major rationale for postnatal corticosteroid therapy.^28^ Currently, there are no markers of lung inflammation in clinical use to identify those infants with elevated inflammatory status and to guide the dose and duration of corticosteroid (or other anti-inflammatory) therapy. In the SASSIE cohort, we previously reported that the cytokines IL-8 and MCP1 were markedly elevated in tracheal aspirates of half of the intubated infants prior to budesonide therapy and that suppression of cytokines with budesonide treatment occurred only in those infants with elevated levels. Using global metabolomics, we further found that there were significant correlations between aspirate cytokine concentration and levels of 24 blood metabolites at baseline, and only 3 metabolites maximally responded to the lowest dose of budesonide: 2 steroids (androsterone sulfate and a hydroxylated pregnanolone derivative) and thiamin (vitamin B1).^17,20^ In the current study with urine, we confirmed that levels of these three lung inflammation-associated metabolites are suppressed by HC in both longitudinal and cross-sectional meta-analyses, with responses (HC post/pre range 0.48–0.86) less than found for lowest-dose budesonide (range 0.12–0.45). Other inflammation-associated biochemicals that were regulated by budesonide were not responsive to HC. While further studies are needed, it does appear that HC at the doses used does not elicit a full anti-inflammatory response, as judged by the putative metabolic markers identified with budesonide, and therefore may be less effective in treating respiratory failure in those infants with lung inflammation.

There are some limitations to our study. The TOLSURF and PROP clinical studies were conducted between 2010 and 2013. Thus, the urine samples were stored (at –70 °C) for up to 10 years and it is possible that oxidation or degradation of some biochemicals may have occurred. However, in a control study, we found a variance of >20% in only 5 of 1191 metabolites after exposure of samples to room temperature for 4 days. The timing of urine collection should not have influenced corticosteroid levels because infants <28-week gestation do not develop a cortisol circadian rhythm for 4–5 months.^29^ Most (93%) of the infants were exposed to maternal antenatal corticosteroid; based on the kinetics of cortisol rebound after antenatal betamethasone, we would not expect any impact at 1 week postnatal age and beyond when the urines were collected.^30^ Some aspects of neonatal clinical practice may have changed from 10 years ago, such as increased use of minimally invasive surfactant administration with non-invasive continuous positive airway pressure;^31^ however, most elements of care remain similar, including the use of HC and TPN. The TOLSURF study protocol included a recommended dosing regimen for HC treatment; however, there may have been instances, based on clinical judgment, where dosing varied from the recommended protocol. All infants in TOLSURF received intravenous TPN beginning at day 0–2 and continuing for 12–46 days before weaning to full oral feeds (primarily breast milk). To avoid the confounding influence of feeding status on metabolite levels,^32^ our study design used only HC-treated infants without a change in feeding status (13 on TPN, 1 on oral feeds) for at least 9 days (range 9–32 days) after starting HC.

In summary, we describe the scope, magnitude, timing, and reversibility of metabolomic changes in infants in response to HC and confirm corticosteroid regulation of three biochemicals that were previously associated with lung inflammatory status. Pending results of additional studies, measurements of specific metabolites of urine or blood (e.g., cortisol and the three markers) may be a useful clinical approach to identifying infants with lung inflammation and for monitoring adrenocortical status and metabolic/inflammatory responses to corticosteroid treatment.

### Supplementary information

Supplementary Table S1

Supplementary Table S2

Supplementary Tables S3 and S4

Supplementary Table S5

## Data Availability

The datasets generated and analyzed during the current study are available from the corresponding author on reasonable request.
