# Supplementary material for: The hydrocortisone-responsive urinary metabolome of premature infants
Source: Pediatr Res. 2023 May 3;94(4):1317–26. doi: 10.1038/s41390-023-02610-5 (PMC10589081; doi:10.1038/s41390-023-02610-5)
Supplement: Supplementary file 3 — Supplementary Tables S3 and S4 [file 41390_2023_2610_MOESM3_ESM.pdf]

Supplemental Table S3. Direction of change by Super Pathway for HC-responsive named metabolites with post/pre value at $p<0.05$

Super Pathway	Decreased Abundance n (% of total)	Decreased Abundance Post/Pre	Increased Abundance n	Increased Abundance Post/Pre
Amino Acid	40 (85.1)	0.67±0.12	7	2.07±0.09
Carbohydrate	17 (100)	0.73±0.10	0	
Cofactors/vitamins	8 (87.5)	0.67±0.06	1	2.49
Energy	6 (100)	0.64±0.08	0	
Lipid	23 (88.5)	0.57±0.19	3	2.12±0.52
Nucleotide	15 (100)	0.75±0.06	0	
Peptide	12 (100)	0.48±0.10	0	
Xenobiotics	11 78.6)	0.64±0.13	3	1.64±0.32

Post/pre abundance is mean/sd of median values for 146 named metabolites in 14 HC-treated infants. n=number of metabolites. 79-100% of metabolites influenced by HC decrease abundance by 27-52%.

Supplemental Table S4. Clinical parameters for infants at age 23-30 days in the cross-sectional analysis of HC treatment.

	TOLSURF			PROP		
	HC	No HC	p	HC	No HC	p
Number of Infants	23	135	-	41	102	-
Maternal Race (Black|Hispanic|White)	15|6|2	66|47|22	0.59	20|14|7	46|40|16	0.32
Gestational Age (wk)	25.0 (1.2)	25.4 (1.2)	0.13	25.4 (1.1)	26.1 (1.1)	0.0009
Male sex (%)	65	57	0.50	54	52	1.00
Birthweight (g)	741 (178)	720 (179)	0.61	737 (148)	804 (146)	0.01
Average RSS d 7-14	4.9 (2.0)	4.0 (1.8)	0.019	2.6 (1.8)	2.6 (1.5)	0.88
BPD at 36 weeks (%)	61	63	0.56	51	51	1.00
On TPN (%)	74	61	0.43	76	52	0.01

Data are mean (sd)
